# Tensions between Western and Indigenous worldviews in pharmacy education and practice: Part II

**DOI:** 10.1177/17151635231188964

**Published:** 2023-07-30

**Authors:** Jaris Swidrovich

**Affiliations:** Leslie Dan Faculty of Pharmacy, University of Toronto, Toronto, Ontario

## Introduction

The first article of this series highlighted how the education and profession of pharmacy is often not a friendly place for Indigenous learners and practitioners and specifically for Indigenous learners and practitioners who see and understand the world through an Indigenous worldview.^1^ Key differences in worldview and knowledge systems were examined between Indigenous and Western intellectual traditions, including the acquisition and dissemination of knowledge. These differences, described as tensions, can and certainly do work together, though. For example, in the practice of braiding together strands of sweetgrass, a certain amount of tension is required to bring each strand together. To achieve an interweaving of knowledge systems, such as Indigenous and Western intellectual traditions, we must formulate a relationship with each tension as we weave them together for the benefit of all. Certainly, Western knowledge systems predominate what is taught and practised in the profession of pharmacy, and so stronger attention must be paid to the unique tensions experienced when weaving in Indigenous knowledges and worldview into Western pharmacy education and practice.

This article is the second in a series of 3 that will elaborate on a few key tensions between Western intellectual traditions and Indigenous knowledges and worldviews. This second part of the series will narrow in on the tensions between each knowledge system with respect to (1) what constitutes evidence and truth and (2) fragmentation and compartmentalization of knowledge and understanding. The third and final article will focus on the lack of regard to land and spirituality in Western pharmacy education and practice. Each of these tensions is offered through critical analyses of the literature and through my own lived and living experiences as an Indigenous (Saulteaux) person who completed pharmacy school and has spent 13+ years as a practising pharmacist.

## Tension #1 in pharmacy education and practice: Evidence-based medicine and what constitutes evidence and truth

Pharmacy education and practice are extraordinarily allegiant to what is referred to as evidence-based medicine (EBM). EBM has been described as “the conscientious, explicit, judicious and reasonable use of modern, best evidence in making decisions about the care of individual patients.”^2^ “EBM . . . requires new skills of the clinician, including efficient literature-searching and the application of formal rules of evidence in evaluating the clinical literature.”^2^ Gazing from and through an Indigenous paradigm, the definition of EBM in and of itself is problematic and oppressive. If EBM truly was “the conscientious, explicit, judicious and reasonable use of modern, best evidence in making decisions about the care of individual patients,”^2^ it would encompass more than Eurocentric Western knowledge systems. The critical, although unnamed, factor of what constitutes EBM is really about who is defining; what is conscientious, explicit, judicious and reasonable; and what is considered to be best evidence.

The phenomenon of Eurocentric Western-based EBM is certainly all powerful. Goldstein and Goldstein^3^ stated that “facts are what all observers agree on.” The institutions of science, pharmacy and medicine are well aware of this and feed the fruits of their knowledge systems to hungry public and governments, desperate and responsible for contributing to the common good. Governments allocate public dollars to major agencies that release calls for research to be produced, such as the Government of Canada’s allocations to the Canadian Institutes for Health Research (CIHR).^4^ The CIHR is considered to be an independent and nonpartisan health research agency that is accountable to the Canadian Federal Minister of Health; however, the CIHR’s mission “to create new scientific knowledge and to enable its translation into improved health, more effective health services and products and a strengthened Canadian health care system”^4^ is entirely rooted in a Eurocentric Western-based paradigm. It was not until 2019 that the CIHR’s Institute for Indigenous Peoples’ Health, and only this 1 institute of 13, allowed nonacademics and research agencies to apply for and hold grant dollars.^4^ As such, researchers funded by the CIHR have been applying (and continue to apply) from and operating within the confines of Eurocentric Western-based paradigms of science and research as they are accountable to the colonial Government of Canada and therefore also to the citizens of Canada. Intuitively, then, the CIHR is wholeheartedly not independent and not particularly nonpartisan; they are dependent on the trust of the public and scientific community and partisan to Western colonial ideas of what constitutes science and research.

Eurocentric Western-based science, to which pharmacy education and practice are strong subscribers, becomes stronger by continuing to fuel public and scientific knowledge and approaches to health care, while at the same time being the sole source of describing what the remaining gaps are in science and health care knowledge. This then prompts public and government bodies to release more public research funds to address such gaps, and the cycle continues. As a result, the literature around pharmacy education and practice, and health care practices in general, continues to be fueled by a single paradigm and knowledge system, which then translates into this single knowledge system and paradigm constituting what is taught and learned in pharmacy education. In addition, as the growing body of “evidence” continues to expand, both public and private dollars turn to funding the outcomes of such evidence. For example, the outcomes of health and pharmacy research, which are grounded in Eurocentric Western ideas of what health and evidence are, inform professional clinical practice guidelines about what constitutes “best” practice. In turn, clinical practice guidelines constitute the “evidence” that inform what governments and insurance companies will consider as fundable and reimbursable health care services, which further erases Indigenous contributions to science and health care.

Currently, with a growing appreciation for equity, diversity and inclusion, we are evolving in our appreciation for other knowledge systems; however, Indigenous and virtually all other practices and knowledge systems that are not rooted in Eurocentric Western science are given an entirely different category, referred to as complementary alternative medicine (CAM).^5^ In pharmacy education and practice, EBM is the underpinning paradigm for all learning, and CAM is “the other.” As such, pharmacy education does not provide the opportunity for learners to even consider Indigenous knowledges as ever being part of EBM, since it has never been part of EBM in the first place.

Within each knowledge system, defining and accepting what is true and what is not true is something of a private club. Goldstein and Goldstein described what they consider to be the duties required to join such a private club:

Often, one cannot judge the truth of some claimed observation without going to the trouble of learning a lot of things that most people do not automatically know. Is the sparkplug removed from the motor of this car burned out or not? This is a question of fact, but not everyone knows offhand how to verify it. One must be not only an observer but also an informed and interested observer.^3^

EBM is, by definition, colonial, and we teach it as the only “truth.” Further, our research funding bodies largely subscribe to this single version of truth.

In a Western science-based educational program and profession such as pharmacy, thinking like a scientist often means that “positivistic notions of scientific knowledge are combined with ontologies of realism and Cartesian duality, to feed on reductionistic and mechanistic practices in order to celebrate an ideology of power and dominion over nature. Science is not value neutral.”^6^ Aikenhead^6^ identifies the positivistic nature of Western science, in that positivism assumes there is a single truth to be found and highlights the conceptualization of the power humans hold over plants and animals; however, these concepts are not shared across all knowledge systems. Pharmacy education and practice subscribe to the same ideology of a single truth, and only Eurocentric Western-based knowledge systems are employed to qualify and quantify evidence, truth and “best practice.” The impetus for all students and professionals in pharmacy, and particularly for Indigenous students and professionals, to think like a Eurocentric Western-paradigm scientist is an ongoing method of colonization where all educational programs and professional practices in pharmacy are complicit. By trying to force a Eurocentric science curriculum on all Indigenous students and professionals, we continue the colonization of the past, a process today called “cognitive imperialism”^7^ and “neo-colonialism.”^8^

Indigenous students must leave their culture at the door and adopt Western approaches to education and curriculum to succeed.^9^ Historically, the academic environment, including pharmacy education, has not enabled Indigenous academic success in any way other than from a Western perspective.^9^ We need to strengthen students’ Indigenous self-identities as they learn to master and critique Western scientific ways of knowing present in pharmacy education without, in the process, sacrificing their own culturally constructed ways of knowing, that is, their Indigenous knowledge.^10^ Throughout our discussions of EBM and in pharmacy education and practice, our aim should be to nurture pharmacy students’ and professionals’ scientific literacy so they can successfully “walk in both worlds,” Indigenous and Euro-Canadian.^11-13^

## Tension #2 in pharmacy education and practice: Compartmentalization of knowledge, health and wellness

Indigenous concepts of knowledge, health and wellness are holistic and interrelated. The concept of the medicine wheel as a representation of health and wellness is well known to Indigenous Peoples. While varying interpretations of the medicine wheel exist, the concepts of mental, spiritual, emotional and physical well-being comprising a person’s whole being is a shared understanding.^14,15^ Whether using a medicine wheel philosophy or otherwise, Indigenous conceptualizations of health and wellness are unlike the compartmentalized and fragmented understandings of health present in the Eurocentric Western paradigm and omnipresent in pharmacy education and practice.

Deloria,^16^ a widely known and respected Indigenous philosopher, discussed the compartmentalization of science in general and noted that our knowledge of the world became “badly fragmented” when the sciences became divided. With the specialization, or fragmentation, of science topics, phenomena and data that do not fit were discarded.^16^ Deloria further elaborated on the discarding of data in specialist subject areas:

Rejected data were called anomalies and no single discipline assumed responsibility for including anomalies in any of the smaller disciplinary paradigms. Thus, there are literally millions of irrefutable facts which science simply dismisses, even though they go to make up entities and events which composed our world.^16^

Despite Western science’s adoption of relativity theories, Deloria postulated that “if scientists *really* believed in the unity and interrelatedness of all things, their emphasis would shift dramatically,” including ceasing the use animals for lab research, adopting considerably different ways of studying water and landscapes and dealing seriously with the by-products of their experiments.^16^

Pharmacy education and the profession of pharmacy in general are rooted in a Western science paradigm and are therefore subjected to the same fragmentation of knowledge, particularly as it relates to science and knowledge about health and wellness. While the concept of holistic health and wellness is present in pharmacy education and practice, it is shared as a stand-alone topic and not as the approach for teaching and learning all topics.^17^ Atleo^18^ affirmed that “the need to focus on isolated variables automatically obscures any assumption about the general nature of interrelationships and connections between variables.” Atleo offered further criticism on the fragmentation of knowledge and isolation of variables in Western science:

. . . if 2 variables are found not to be related, does this language not indicate that the universe is fragmented according to scientific criteria? Not only does this use of language, “not significantly related,” reflect a scientific view of existence, but it is also reflected generally in the fragmentation of Western thought – in the separation of church and state, for example – and furthermore in Western policies and practices.^18^

While the Western science that fuels pharmacy education indeed yields powerful insights into isolated fragments, the sum of these insights is a disconnected, inadequate description of the whole.^19^ Indigenous worldviews, however, always assume a meaningful relationship between variables.^18^ In addition, Indigenous worldviews may never be completely substantiated because of human limitations, which is an accepted reality, unlike Western intellectual traditions that will repeatedly attempt to reduce knowledge into variables and describe the relation, or lack of relation, between them. Consider an example offered by Goldstein and Goldstein^3^ regarding studying the cause(s) of lung cancer. After identifying as many people with lung cancer as possible and an equal number of people who do not have the disease, Western-based scientists will list the circumstances attending each case: age, sex, employment, ethnic origin, smoking history, dietary habits, income, the number, age and sex of children, marital status, neighbourhood lived in, type of house lived in, number of rooms, type of furniture, street, age of parents and cause(s) of their death, details of education, model of car driven, likes and dislikes in books and music and more.^3^ It quickly becomes apparent that the list has a tremendous number of individual details and with virtually no end. Goldstein and Goldstein suggested that if one objects that most of the details in the list are irrelevant, the answer must be, “How do we know?” and that “it is only because we already have some feeling, even if hazy, as to possible causes of cancer that we can rule out most of the detailed circumstances of the list.”^3^ Goldstein and Goldstein^3^ concluded that “many of the great scientific discoveries resulted from recognizing the relevance of facts that were previously overlooked and putting aside facts previously considered important,” which sheds light on the interconnectedness of all things.

**Figure 1:**
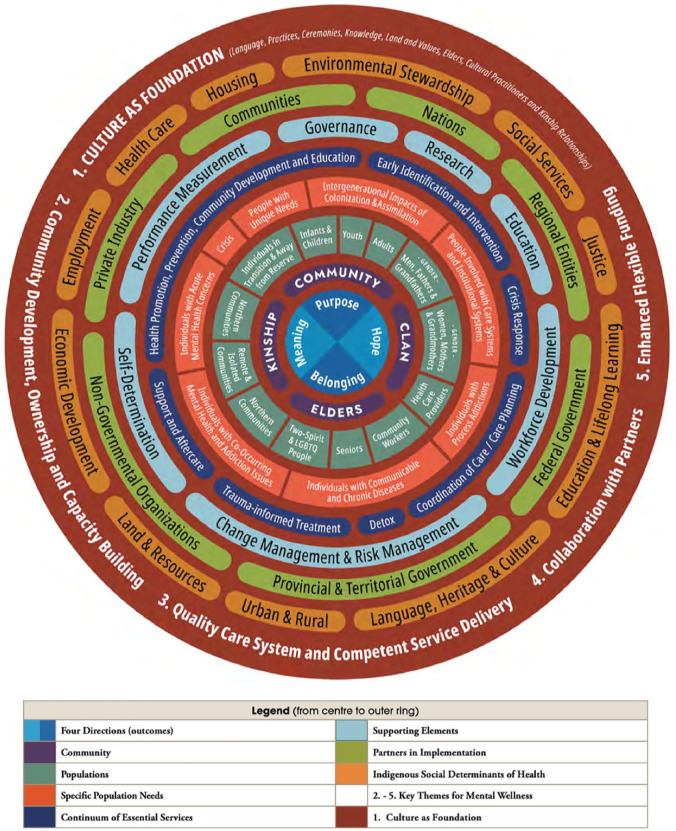
First Nations mental wellness continuum model^21^

When it comes to teaching and learning in pharmacy education and practice, concepts of health and wellness are compartmentalized into various human body systems and disease states. When holistic approaches are examined, as well as critical evaluation of the social determinants of health and wellness, it is often performed in an independent way and outside of the context of each individual human body system and/or disease state. Learning outcomes and accreditation standards for pharmacy education in Canada do not necessitate teaching and learning about health and wellness through holistic and social determinant approaches and instead only specify teaching about holistic and social determinant approaches.^17,20^ As such, both teachers and learners in pharmacy education are individually responsible to determine what, if any, connections can be made between human body systems and/or individual disease states and other external (e.g., social, environmental, political, ecological, etc.) factors, if ever. Through an Indigenous lens, the Thunderbird Partnership Foundation created a First Nations Mental Wellness Continuum Model to showcase all the interrelated factors to consider in someone’s health and wellness, which is much more comprehensive than the biomedical model used in pharmacy education and practice, which may or may not make reference to a smaller set of social determinants of health (Figure 1). As a result, teaching and learning in such compartmentalized and fragmented ways continues to privilege and perpetuate Western understandings and approaches to health and wellness in pharmacy education and practice.

## Conclusion

Each of the 2 tensions discussed provide an opportunity for the reader to appreciate the perspectives of Indigenous Peoples within pharmacy education and the profession of pharmacy. Recognizing that the predominant worldview present within pharmacy education and practice is Western, these tensions exclusively focused on the Indigenous perspectives of various content and have been described from the point of view of an Indigenous person within the educational and professional structures of pharmacy. The overall approach to education and practice, however, does not have to be one or the other but rather a weaving of multiple knowledge systems. We must formulate a relationship with these tensions and (re)consider them as we weave together Indigenous and Western intellectual traditions across the education and professional landscapes of pharmacy. After all, Indigenous Peoples are our patients, they are our educators, and they are our pharmacists and all other pharmacy professionals. Please, walk with the teachings within this article and we will gather again for the third and final article of this series. This, my friends, is reconciliACTION.
